# Quality compliance in the development of cell-based medicines in non-pharma environments

**DOI:** 10.1186/1753-6561-9-S9-P29

**Published:** 2015-12-14

**Authors:** Irene Oliver-Vila, Amy L van Deusen, Roger Palau, Joaquim Vives

**Affiliations:** 1 Divisió de Teràpies Avançades/XCELIA, Banc de Sang iTeixits, Edifici Dr. Frederic Duran i Jordà, Passeig Taulat, 116, 08005 Barcelona, Spain; 2 University of Virginia, Department of Pharmacology, Charlottesville, 22901,VA, USA

## Background

Academic institutions and transfusion centres are positioned to lead the development of novel cell-based therapeutics up to early-stage clinical trials (and further seeking marketing authorisation) or, alternatively, its use under the hospital exemption clause [1,2]. Existing structures, including blood and tissue bank facilities, translational medical programs and skilled personnel experienced in cell culture technologies and transplantation, equips these non-commercial entities to translate research-grade cell materials into safe and commercially viable products suitable for human use. However, developers must negotiate the complex regulatory and quality requirements involving the compulsory compliance of Good Scientific Practice (GxP) regulations in the development of advanced therapy medicinal products (ATMP) [3]. Originally designed for corporate environments, implementation of GxP in non-pharmaceutical institutions may consume a considerable level of effort (particularly in terms of funding and human resources). In non-pharmaceutical environments, current quality management structures for cell therapies are highly specific for blood and bone marrow-related products, including programs administered by JACIE, NetCord, FACT, AABB, or focused upon more global quality objectives, such as ISO guidelines (particularly ISO9001). The adaptation of such either general or specific quality assurance programs into GxP may appear as a substantial effort. However, many of the requirements are common across standards and the existence of any of them in a given institution can suit up to some extent to some of the aspects covered by the GxP. Rather than focusing on each certification separately, product developers and manufacturers in all environments would benefit from a single quality management system incorporating the most convenient tools available from different standards. In the present work, we analysed the GxP requirements and those in voluntary quality accreditation schemes already present in any blood and tissue bank. A number of common aspects were found, therefore offering an opportunity to avoid duplicities in the implementation process. Our work aims at serving as a guide for the optimisation of resources available and contributes to moving towards a single quality assurance system that integrates others, thus improving efficiency.

## Materials and methods

Generic quality assurance systems (such as International Organization for Standardization, ISO, www.iso.org, guidelines, particularly ISO9001), and specific quality assurance systems for blood and bone marrow-related implemented in the Banc de Sang iTeixits in Barcelona (that is The Joint Accreditation Committee-ISCT Europe & EMBT, JACIE, www.jacie.org; NetCord, www.netcord.org; and Foundation for the Accreditation of Cellular Therapy, FACT, www.factweb.org) but also the American Association of Blood Banks (AABB, www.aabb.org) were analysed and compared to Good Manufacturing [4-7] and Laboratory [8-10] Practice (GMP and GLP, respectively) regulations.

## Results

**Figure 1:**
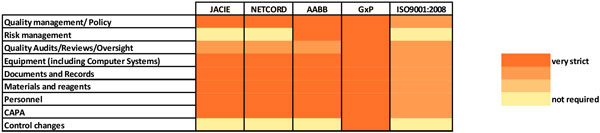
**Compatibility of quality standards for cellular therapies**. The cross-comparison of different quality management systems used in the development and production of cellular therapeutics and ISO9001:2008 highlights GxP as the strictest system. However, similarities among standards and the versatility of ISO9001:2008 offers potential ways to simplify common processes.

The implementation of GxP in our institution was performed gradually along the development programme of ATMPs composed of Bone Marrow-derived Mesenchymal Stromal Cells (BM-MSC) aiming at articular cartilage and bone regeneration [11]. In order to ensure the coordination with other standards already present in our institution, we analysed their compatibility by cross-comparison, resulting in the heat map presented in Figure 1. Not surprisingly, GxP emerged as the strictest quality management system, although similarities among standards were unveiled and, remarkably, the versatility of ISO9001 showed potential solutions for simplifying common processes, as discussed next.

• *Quality management/Policy: *A single Quality Review and Quality Plan can be formulated from the ISO9001 perspective and suit all other standards.

• *Risk management: *Risk management is mandatory for AABB and GxP, and represents an excellent tool that can be applied to other standards, thus facilitating the detection of critical aspects requiring changes. Besides, risk management will be incorporated as a new requirement in next ISO revision (ISO 9001:2015).

• *Quality Audits/Reviews/Oversight: *Rather than audit plans for each standard, it is recommended to have a single plan approved by the general manager. This audit plan must be developed to address the specific points of each standard. Moreover, significant attention to personnel performing audits is recommended, as ongoing training and competence according to each individual standard is required. A pool of auditors, comprised of individuals with different competences, is most effective in this regard.

• *Equipment (including Computer Systems): *It is recommended to create and maintain a single database for the control of equipment. While this aspect varies widely across standards, GMP emerges as one of the strictest, so more information for equipment present in GMP facilities must be included and maintenance performed accordingly (Installation/Operational/Performance Qualifications in addition to calibration/verification).

• *Documents and Records: *A single document manager for the whole company is recommended, provided that some standards are very strict in having paper register of all documentation, we suggest to adopt the strictest regulation, in our case is governed by the GLP, in particular with respect to archiving. This has a substantial impact on the amount of documentation existing in the organisation.

• *Materials and reagents: *potential to unify processes for planning and controlling all steps in the acquisition and use of goods. In this sense, the use of control guidelines is essential for a close control of the specifications of raw materials and reagents.

• *Personnel: *In order to avoid each division/department/unit qualifying their own personnel, it is proposed to manage this aspect for the institution as a whole, by using a single system fulfilling all applicable standards.

• *Corrective Action/Preventive Action (CAPA): *The main recommendation here is to generate a single database to manage "no conformities" and a quality assurance unit that validates them and follows up. All personnel must be trained on how to use relevant systems and databases for managing deviations and incidents.

• *Control changes: *this is a useful tool in any quality system that we apply also to other processes within the company in order to ensure a better practice in our "day-to-day" activities. This tool requires considerable initial effort from personnel involved, provided that changes must consider why, how, whom.

Our analysis unveils the benefits of the adoption of a single system that integrates the quality management structures for different types of cell therapies (both traditional cell transplants and ATMP). Indeed, a single quality assurance unit supporting all certifications would become less specific for blood and bone marrow-related products, and more open to future cell-based therapies (Table 1).

## Conclusions

Both the development of novel ATMP and the improvement of quality standards in traditional blood-related products would likely drive broader adoption of GxP. Cooperative efforts from different departments in the same institution aiming at reducing duplicities and ensuring consistent application of quality programs would contribute to improve efficiency.
